# Using a Vibration Device to Ease Pain During Facial Needling and Injection

**Published:** 2016-02-04

**Authors:** Hiroaki Kuwahara, Rei Ogawa

**Affiliations:** ^a^Department of Plastic and Reconstructive Surgery Aidu-Chuo Hospital, Fukushima, Japan; ^b^Department of Plastic, Reconstructive and Aesthetic Surgery, Nippon Medical School Hospital, Tokyo, Japan

**Keywords:** gate control theory, vibration, pain, face, the trigeminal nerve

## Abstract

**Objective**: In general, needling and injection are painful procedures, especially when the face is the target. Although local anesthetics (cream or tape) can be used to reduce the pain, they are not sufficiently effective. It has been suggested that vibration can reduce pain. The aim of this case study was to determine whether application of a vibration device to an area adjacent to the facial target area to be injected/needled would relieve pain. **Methods:** Consecutive women scheduled to undergo facial injection with hyaluronic acid or botulinum toxin were recruited. Half of the face was injected with concomitant vibration, whereas the other half was injected without vibration. The pain experienced by the women during both procedures was assessed using the Numeric Rating Scale. The safety of injection with vibration was also assessed. **Results:** Of the 32 patients, 28 indicated that vibration relieved the pain, 3 stated that it had no effect, and 1 (who received deep botulinum toxin injections to the masseter muscle) complained that it made the pain worse. Vibration did not affect the safety of the injections. The average Numeric Rating Scale scores for the no-vibration and vibration injections were 4.5 ± 1.5 and 2.3 ± 0.9, respectively (*P* < .001). **Conclusions:** The Gate Control Theory of Pain explains why vibration reduces pain.

Injections cause patients anxiety and stress, especially when the target is the face. Health care professionals should strive to reduce injection-induced pain as much as possible. Various methods of relieving injection-related pain have been proposed, including icing, application of cool air, performing the injection slowly, and iontophoresis.^1^ Recently, however, several reports suggest that a vibration device effectively relieves injection-associated pain and is safe.^2^^-^7

It is likely that vibration devices relieve pain because they induce stimulation-induced analgesia. The concept of stimulation-induced analgesia was proposed many years ago and originates from the Gate Control Theory of Pain that was first reported in 1965 by Melzack and Wall. This theory states that when A-β fibers (large fibers) are stimulated by non-noxious stimuli, they prevent pain signals transmitted by A-δ or C fibers (small fibers) from reaching the central nervous system. As a result, the non-noxious stimulus suppresses pain.

To determine whether vibration devices effectively and safely relieve facial pain, the present case study was performed. In all cases, half of the face was injected simultaneously with the application of a simple noninvasive vibration device and the other half was injected without vibration.

## METHODS

The case series comprised consecutive female patients who received hyaluronic acid or botulinum toxin facial injections in the Department of Plastic and Reconstructive Surgery at Aidu-Chuo Hospital from 2014 to 2015 and agreed to participate in the study. The study was approved by the Aidu-Chuo Hospital Ethics Committee, and informed consent was obtained from all patients.

**Figure 1:**
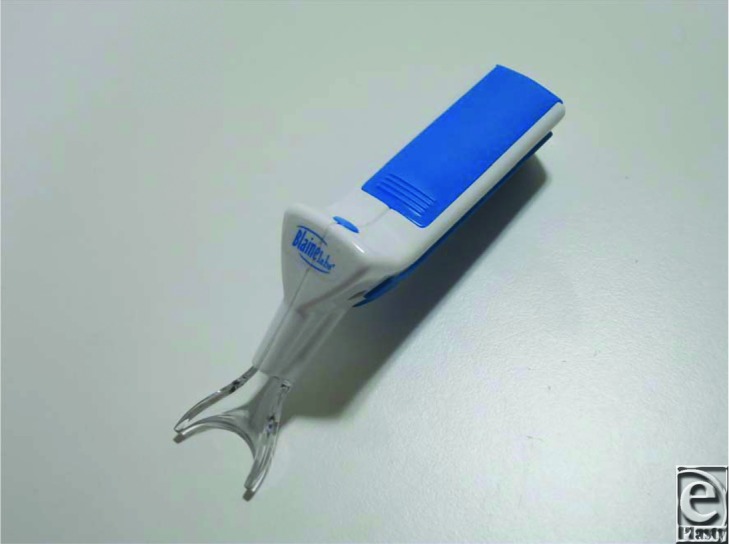
Vibration Anesthesia Device (Blaine Labs, Inc).

**Figure 2:**
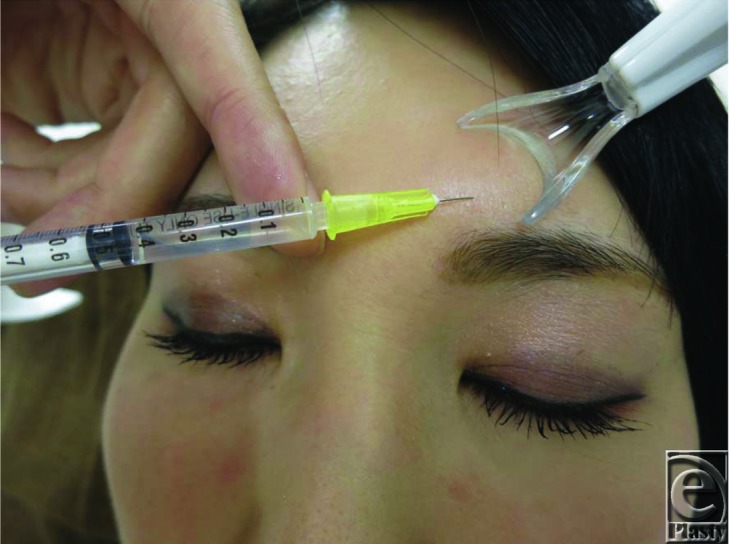
One-half of the face was injected with hyaluronic acid or botulinum toxin with concomitant application of the vibration device. The other side was injected without vibration. Vibration stimulus is comparatively strong, attaching the hand is recommended.

For all patients, one-half of the face was injected with hyaluronic acid (Restylane) or botulinum toxin (Juvederm Vista) with concomitant application of the Vibration Anesthesia Device at 150 at 183 Hz (9000-11,000 times per minute) (Blaine Labs, Inc, Santa Fe Springs, CA) (Fig 1). The other side was injected without vibration (Fig 2). No patients had received any pretreatment. During needling/injecting, the device was used from beginning to end. However, it was avoided when making a smooth outline formation such a tear bag. Whether the patient was injected with or without vibration in the first half of the treatment was determined by using block randomization method. Thus, the area adjacent to the injection/needling site contacting with the device received continuous vibration stimulus during the injection before the injections were performed. The device does not interfere in an injection part because of it is Y-shaped.

After each side was injected, the patient was asked to estimate the degree of pain on the Numeric Rating Scale (NRS).

## STATISTICAL ANALYSIS

The Wilcoxon signed-rank test was used to assess whether vibration significantly influenced the pain caused by facial injections/needling. Confidence intervals were set at 95%. All statistical analyses were performed using StatMate IV software version 4.01 (Advanced Technology for Medicine and Science, Tokyo, Japan).

## RESULT

In total, 32 women were enrolled in the study. The average age was 43.0 (range, 28–73) years. Twenty patients received hyaluronic acid injections, and the remaining 12 patients received botulinum toxin injections.

The trigeminal nerve (also called cranial nerve V) is 1 of 12 pairs of cerebral nerves. This trident-shaped nerve is divided into 3 branches: ophthalmic nerve (V1), maxillary nerve (V2), and mandibular nerve (V3). Analysis of the injection sites revealed that the majority were in areas served by the V1 and V2 nerves. Thus, 6 patients receiving hyaluronic acid underwent V1 injections: 3 were injected at the wrinkle at the root of the nose, 1 was injected in the frontal region of the head, and 2 were injected during rhinoplasty of the nose root. Twelve patients receiving hyaluronic acid underwent V2 injections: 9 were injected at the nasolabial fold, and 3 were injected at the eye (tear) bags. Two patients receiving hyaluronic acid underwent V3 injections at the marionette line. Of the patients receiving botulinum toxin, 8 underwent V1 injections: 3, 1, and 4 were injected at the frontalis, procerus, and supercilii muscles, respectively. Three patients receiving botulinum toxin underwent V2 injections at the side and lower eyelid, and 1 underwent V3 injections into the masseter muscle.

**Figure 3:**
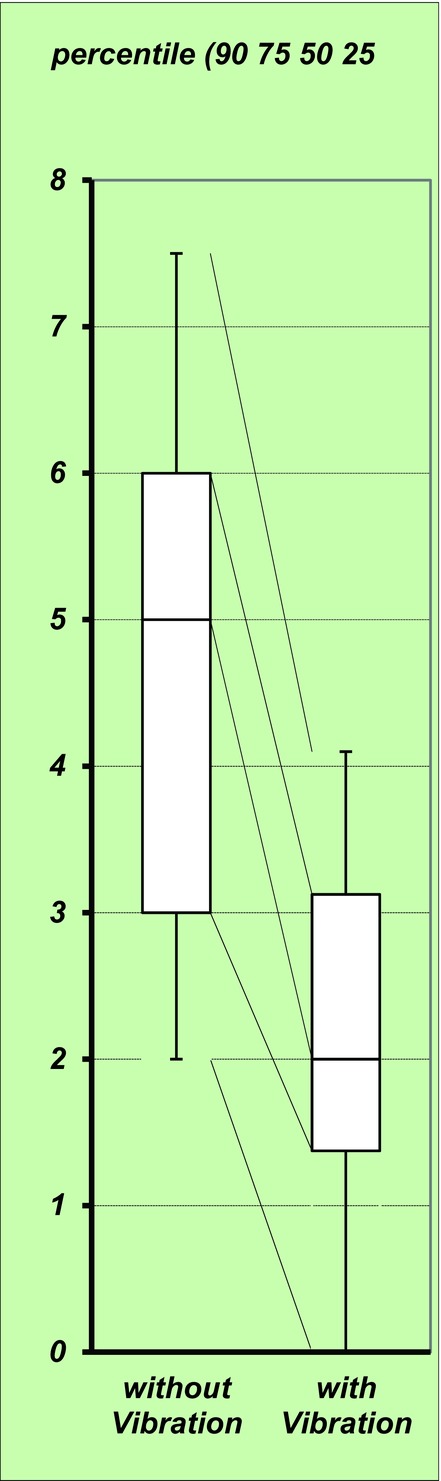
The average Numeric Rating Scale scores for the no-vibration and vibration injections were 4.5 ± 1.5 and 2.3 ± 0.9, respectively (*P* < .001).

The average NRS scores for the no-vibration and vibration injections were 4.5 ± 1.5 and 2.3 ± 0.9, respectively (*P* < .001) (Fig 3). Of the 32 patients, 28 reported pain relief during vibration (compared with the pain associated with injection of the nonvibrated side). Of the remaining 4 patients, 3 reported that vibration did not alter the pain whereas 1 (the patient who received botulinum toxin injections at the masseter muscle) complained that the vibration made the pain worse.

## DISCUSSION

Although it is widely recognized that creams, tape, and injection effectively generate local anesthesia,^8^^,^^9^ there is some concern that they may induce allergy and dermatitis^10^ or other risks. This is particularly true when they are used to induce nerve block (eg, axillary nerve block), which is required for laser treatment of a large region or botulinum toxin treatment of hyperhidrosis. Moreover, local anesthesia injections cannot be used to relieve the pain caused by facial injections because the anesthesia injections themselves cause pain. Various methods of reducing injection-associated pain have been considered. Recently, it was suggested that application of a vibration device effectively relieves the pain associated with dermatological procedures.^2^^-^7

The present case series confirmed that vibration effectively relieves injection-induced pain: 28 of 32 patients reported that they experienced less pain when vibration was used. Three of the remaining patients stated that they experienced no difference. Only 1 patient reported that vibration made the pain worse.

**Figure 4:**
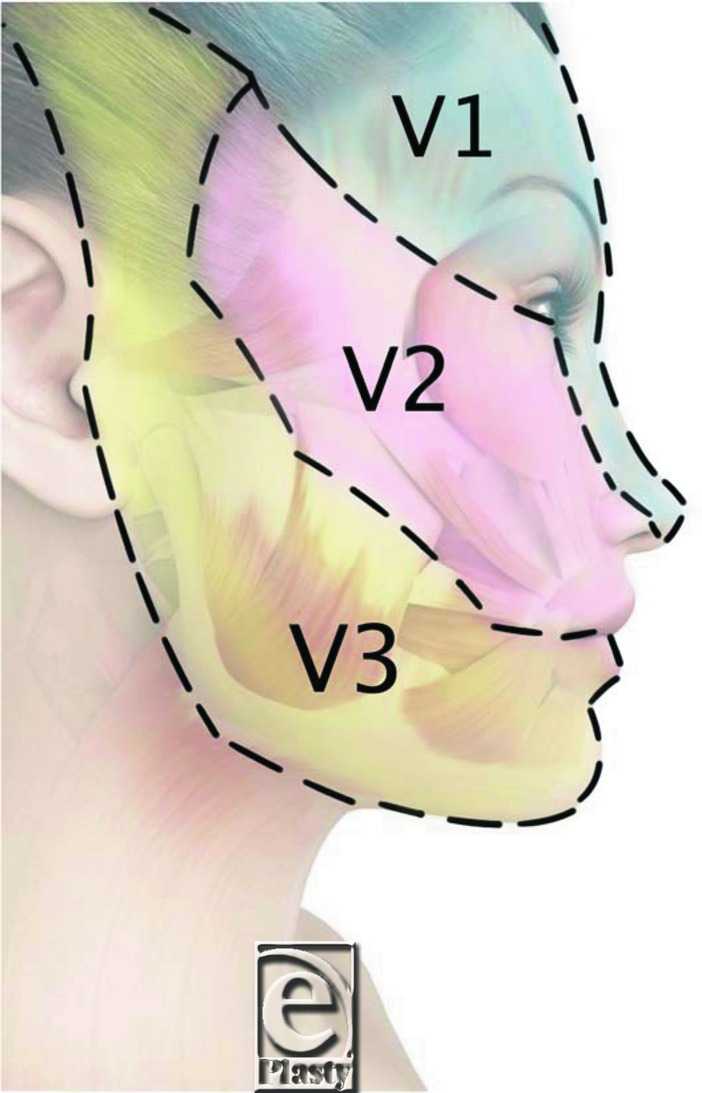
Territories of the trigeminal nerve: Ophthalmic nerve (V1); maxillary nerve (V2); and mandibular nerve (V3).

The trigeminal nerve is the largest of the cranial nerves and is responsible for motor function and sensation in the face. Its V1 branch is distributed over the back side of the eye orbit after passing the superior orbital fissure; it is therefore predominantly responsible for sensation perception in the forehead, upper eyelid, and nose. The V2 branch is mainly responsible for sensation perception in the upper jaw, upper gum, palate, and lower eyelid. The V3 branch is predominantly responsible for sensation perception in the jaw, lower lip, lower gum, and part of the ear lobe (Fig 4). In our case series, the majority of facial injections affected the V1 and V2 branches; only 3 were injected in V3 locations. These included the patient who complained that vibration made the pain worse. This patient received botulinum toxin injections into the masseter muscle. Since this was also the deepest operation of the case series, it may be that the effect of the vibration was weakened by the depth. To resolve this, further assessment of the effect of vibration in additional cases of V3 operations is warranted.

The NRS was selected to estimate the pain associated with the injections. This is an 11-point scale on which “0” indicates no pain and “10” indicates the worst pain imaginable. It was selected because it is both accurate and easy to use. Alternative tools include the visual analog scale, which is used most commonly in clinical settings. However, it has the disadvantage that it requires the use of a writing implement or a scale device. Moreover, it is reported that the visual analog scale is inferior to other scales used for clinical evaluation.^11^ Another alternative is the Verbal Rating Scale; however, this scale may be hampered by linguistic problems. Moreover, its objectivity is limited.

The Gate Control Theory of Pain (first proposed by Melzack and Wall in 1965) may explain why the vibration device relieved the pain associated with facial injections. This theory states that non-noxious stimulation of the spinal cord via somatic sensation closes the “gate.” There are the 2 types of neural fibers; namely, the small-diameter A-δ and C fibers and the large-diameter A-β fibers. The latter fibers sense vibration. The neural fibers carry information to the central nervous system via the dorsal horn of the spinal cord. The small fibers transmit pain stimuli to the brain, whereas the large fibers inhibit the firing of small fibers (ie, they close the “gate”).^12^^,^^13^ This mechanism explains why a non-noxious stimulus such as vibration can suppress pain.

Many studies report that devices with vibratory function reduce the pain associated with dental and cosmetic procedures as well as incision and drainage; they can also reduce needlephobia.^2^^-^4 Furthermore, vibratory devices have been used during venipuncture in infants^14^ and to treat diabetic peripheral neuropathy.^15^ The present case series also suggests that vibration may provide pain relief in almost all instances of facial injection. However, there are some limitations to this method. First, while our patients did not dislike the vibration stimulation itself, several did complain about it at certain stages of the operation. One commented that “the pain was reduced but needling with vibration feels like a tattoo is being carved.” Another complained that she “felt the vibration was hard, especially on areas where bone was projecting.” Second, while we did not observe that vibration during injections had harmful side effects, we recommend clinicians avoid using a vibration device when making a smooth outline formation, such a tear bag, or when injecting a risky area, where the vibration could cause liquid medicine to spread into the orbit. Also, vibration should not be used when the needle must be kept in the dermis. Thus, while a vibration device can be used during needling, it should be avoided during the aforementioned injections.

Despite the overall efficacy of the vibration device, very few of our patients said that it caused needling and injection to become completely painless. Since there is no device to date that can remove pain completely, further research on how to reduce patient anxiety associated with needling and injection is warranted.
